# The Recent Progress in Nanotoxicology and Nanosafety from the Point of View of Both Toxicology and Ecotoxicology

**DOI:** 10.3390/ijms21124209

**Published:** 2020-06-12

**Authors:** Yuan-Hua Wu, Sheng-Yow Ho, Bour-Jr Wang, Ying-Jan Wang

**Affiliations:** 1 Department of Radiation Oncology, National Cheng Kung University Hospital, College of Medicine, National Cheng Kung University, Tainan 704, Taiwan; wuyh@mail.ncku.edu.tw; 2 Department of Radiation Oncology, Chi Mei Medical Center, Tainan 736, Taiwan; shengho@seed.net.tw; 3 Graduate Institute of Medical Sciences, Chang Jung Christian University, Tainan 711, Taiwan; 4 Department of Cosmetic Science and Institute of Cosmetic Science, Chia Nan University of Pharmacy and Science, Tainan 71710, Taiwan; 5 Department of Occupational and Environmental Medicine, National Cheng Kung University Hospital, Tainan 704, Taiwan; 6 Department of Environmental and Occupational Health, College of Medicine, National Cheng Kung University, Tainan 704, Taiwan; 7 Department of Medical Research, China Medical University Hospital, China Medical University, Taichung 404, Taiwan

## Abstract

This editorial aims to summarize the 14 scientific papers contributed to the Special Issue “Nanotoxicology and nanosafety 2.0 from the point of view of both toxicology and ecotoxicology”.

Recently, nanotechnology has been rapidly promoting the development of a new generation of industrial and commercial products and areas such as catalysts, sensors, environmental remediation, personal care products and cosmetics. In addition, nanomaterials also show great promise in the field of medicine in application such as imaging and drug delivery. However, the hazardous impact of nanoscale particles (NPs) on organisms have not been thoroughly clarified yet. In some cases, nanomaterials present unexpected risks to both humans and the environment. Assessments of the potential hazards associated with nanotechnology have been emerging, but substantial challenges remain because the huge amount of different nanomaterials cannot be effectively evaluated in a timely manner [1,2,3]. Although there exist numerous approaches to perform toxicity tests, common and reasonable biomarkers for toxicity evaluations are still lacking and need more investigation [4,5].

From the toxicological point of view, in terms of cytotoxicity, genotoxicity, hepatotoxicity, and embryo toxicity both in vitro or in vivo the toxic effects induced by metal or metal oxide NPs, such as AgNPs and ZnONPs are quite similar [4]. NPs-induced oxidative stress was considered as one of the initiators that can trigger disruption of mitochondrial membrane potential and induction of apoptosis and/or autophagy. It has been reported that AgNPs can induce ROS generation and apoptotic cell death in NCM460 and HCT116 human colon cells through regulation of p38, p53, Bax/Bcl-2 ratio and P21 [6]. Most in vitro studies have demonstrated the size- and surface coating-dependent cellular uptake of AgNPs. In vivo bio-distribution studies of AgNPs indicated that AgNPs could be accumulated in local as well as distant organs [7]. Intragastric exposure of mice to ZnONPs for 28 days disrupted the seminiferous epithelium of the testis and decreased the sperm density in the epididymis. Both apoptosis and autophagy can be induced in the testis tissues, meanwhile, up-regulation of the cleaved Caspase-8/Caspase-3, Bax, LC3-II, Atg 5, and Beclin 1 were found too. In vitro tests showed that ZnONPs could induce apoptosis and autophagy with the generation of oxidative stress in mouse Leydig cells [8]. ZnONPs were internalized into cells in both particle and ion form and was transported through intestinal barriers and absorbed in the small intestine primarily as Zn ions, whereas, a small amount of ZnO was absorbed as particles [9].

Toxic effects of nickel oxide (NiO) and nickel hydroxide (Ni(OH)_2_) nanoparticles were analyzed in human lung (A549) and hepatocellular carcinoma (HepG2) cell lines. The results indicated nickel NPs were toxic to A549 cells but relatively nontoxic to HepG2 cells. Cytotoxicity was mediated by oxidative stress-induced apoptosis and suppression of cell proliferation [10]. In addition, transition metal oxide NPs including TiO_2_, Cr_2_O_3_, Mn_2_O_3_, Fe_2_O_3_, NiO, CuO, and ZnO were analyzed in the same A549 cell culture model and the authors found that all NPs aside from Cr_2_O_3_ and Fe_2_O_3_ showed a time- and dose-dependent decrease in viability. The trend of cytotoxicity was in parallel with proliferative inhibition, revealing a strong correlation among viability, proliferation and apoptosis [11].

NPs may enter human body via various routes such as ingestion, inhalation and skin contact. After translocation with the help of the tblood and lymph systems, they are transported into other organs and lead there to systemic effects [12]. When considering the skin contact route, understanding the uptake mechanisms of ionic, elemental or metal NPs in combination with bioactive substances are important for the assessment of related health impact. High aluminum (Al) intake could lead to neurotoxicity and triggers concern about potential health risks such as Alzheimer’s disease. Al NPs in cosmetic products raise the question about whether a possible interaction between Al and vitamin A/D metabolism might exist. Thus, the uptake and distribution of Al oxide (Al_2_O_3_) and metallic AlO NMs in the human keratinocyte cell line HaCaT were conducted. The results indicated that vitamin A and D exposure of cells affects the intracellular uptake of Al NPs and its agglomeration behavior [13]. When considering the inhalation route, systemic toxicity in rats after a short duration inhalation of lead oxide nanoparticles has been reported. Rats were exposed to lead oxide nanoparticle aerosols during 5 days for 4 h a day in a nose-only setup, demonstrating a lead oxide NPs retention in the lungs and the olfactory brain. Several lead-specific toxicological outcomes were found in the exposed rats, those included increase in reticulocytes proportion, δ-ALA urine excretion and the arterial hypertension’s development [14].

From the ecotoxicological point of view, various NPs from industry might present possible risks to aquatic systems. Besides the metal or metal oxide NPs, carbon NPs such as fullerene (C_70_) and nanoplastics has drawn great attention. Nevertheless, the comprehensive biological and toxicological effects of those NPs on the environment has still not been studied in detail, thus, it is important to analyze the effects of NPs on ecological systems as well as human health. The zebrafish test is an appealing in vivo model to assess the hazards of both conventional chemicals and NPs in ecotoxicology [15,16,17]. Identification of the targets and mechanisms for the deleterious effects of AgNPs in a zebrafish embryo model has been conducted. Exposure of AgNPs led to lysosomal activity changes and higher number of apoptotic cells distributed among the developmental organs of the zebrafish embryo. AgNPs of larger size exhibited different behavior from the smaller size, in which the embryo chorion play a pivotal role [15].

The toxicity of C_70_ NPs in lung and skin cells have been demonstrated, whereas, the potential detrimental role in neurobehavior is largely unknown. Chronic effects of C_70_ NPs exposure for two weeks on behavior and alterations in biochemical responses in adult zebrafish were performed. The results indicated a decreased locomotion, exploration, social interaction, mirror biting, as well as anxiety elevation and circadian rhythm locomotor activity impairment. Regarding the biochemical assays, the activity of superoxide dismutase (SOD), reactive oxygen species (ROS) and cortisol in the brain and/or muscle tissues increased significantly [17].

Currently, microplastics and nanoplastics are everywhere, contaminating our water, air, and food chain [18]. The toxicity of microplastic exposure on humans and aquatic organisms has been documented, but data on the toxicity and behavioral changes of nanoplastics in mammals are scarce. Polystyrene nanoplastics (PS-NPs) was applied to investigate the neurobehavioral alterations, tissue distribution, accumulation and specific health risk of nanoplastics in adult zebrafish. The results demonstrated that PS-NPs accumulated in the gonads, intestine, liver, and brain and induced disturbance of lipid and energy metabolism as well as oxidative stress and tissue accumulation. Chronic exposure of high dose PS-NPs induced behavior alterations in their locomotion activity, aggressiveness, shoal formation, predator avoidance behavior and dysregulated circadian rhythm locomotion activity [16]. Thus, both embryo and adult zebrafish toxicity assays are suitable for investigating the metal and carbon-based NPs’ ecotoxicologial effects.

Alternative testing strategies are commonly used to assess the safety of chemicals, and many of these strategies were evaluated for the testing of NPs. In vitro testing was proposed as the principal approach with the support of in vivo assays to fill knowledge gaps, including tests conducted in non-mammalian species such as drosophila and zebrafish [3]. Human cell-based systems are recommended as relevant models to reduce uncertainty and to improve prediction of human toxicity. When compared with the transformed or immortalized cell lines, primary cells have similar characteristics as the original donor tissue, so more and more scientists are considering the use of primary cells in in vitro studies to establish more biologically representative models [19]. Recently, stem cell-derived models are under investigation as potential models for nanotoxicity assay. Stem cells can offer the opportunity to model the reproducibility of rare phenotypes in vitro [20]. For example, neuronal toxicity of Fe_3_O_4_ NPs has been demonstrated by using the in vitro differentiation of the human umbilical cord lining-derived-mesenchymal stem cells (hCL-MSCs) into neuron-like cells (hNLCs) as an ideal primary cell source of human origin [21]. The study demonstrated that hCL-MSCs can be easily differentiated into neuronal-like cells and the hNCLs are susceptible to Fe3O4NPs, thus, human primary cultures of neurons could be a new in vitro model for NPs evaluation [21].

A thorough understanding of the mechanisms by which NPs perturb biological systems is critical for a more comprehensive elucidation of their nanotoxicity and will also facilitate the development of prevention and intervention policies against adverse outcomes induced by NPs. The development of a good strategy for NM hazard assessment not only promotes a more widespread adoption of non-rodent or 3Rs principles but also makes nanotoxicology testing more ethical, relevant, and cost- and time-efficient. We hope that the abovementioned articles can provide updated knowledge regarding the nanotoxicology and nanosafety from the point of view of both toxicology and ecotoxicology.
